# Ferrocenes as Building Blocks in Molecular Rectifiers and Diodes

**DOI:** 10.3390/molecules23071551

**Published:** 2018-06-27

**Authors:** Mark E. Welker

**Affiliations:** Department of Chemistry, Center for Functional Materials, Wake Forest University, 455 Vine Street, Winston-Salem, NC 27101, USA; welker@wfu.edu; Tel.: +1-336-702-1953

**Keywords:** diode, ferrocene, molecular rectifier, rectification, self-assembled monolayer, unidirectional charge transfer

## Abstract

Ferrocenes have recently been reported as components of a number of molecular circuits. This short review covers reports of ferrocenes in molecular rectifiers and diodes which have appeared in the last 10 years.

## 1. Introduction

As the length scales relevant to commercial electronics continue to shrink, great effort has been expended to explore the feasibility of devices with dimensions approaching that of a single molecule [1,2,3,4,5,6,7,8]. The molecular junctions, or molecular diodes, represent the simplest devices based on a single molecule or an array of molecules sandwiched between two electrodes. In these devices, the current versus voltage behavior is highly dependent on the polarity of the applied bias, i.e., they can rectify the current, similar to a p–n junction. The magnitude of rectification depends on the structure and order of the monolayer, as well as the choice and quality of contacts. In this review we cover recent reports of ferrocene-containing molecules which can perform as molecular rectifiers. When incorporated in molecular rectifier devices, these compounds perform the same function as a diode in which current is allowed to pass through at one bias while a significantly decreased current passes at the opposite bias.

## 2. Discussion of Prior Work

The format of this review is largely chronological, discussing work involving ferrocenes as components of rectifiers or diodes from 2008 to 2018. In some instances, work of one group is presented chronologically within that same context before moving on to the work of another research group. Prior computational/theoretical work on ferrocene-containing rectifiers will be presented first in Section 2.1, followed by experimental work of the last ten years on ferrocene-containing rectifiers in Section 2.2.

### 2.1. Computational/Theoretical Work on Ferrocene-Containing Rectifiers

**Scheme 1:**
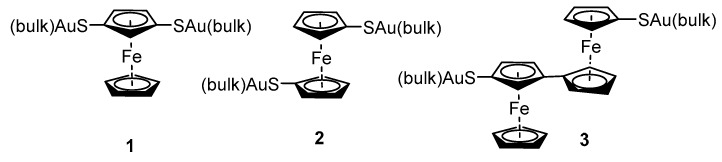
Ferrocene dithiolates bonded to Au.

In 2008, Mizuseki and co-workers examined electronic structures of a number of ferrocenes using density functional theory [9]. These structural calculations were accompanied by an investigation of their electron transport properties using nonequilibrium Green’s function formalism for quantum transport. Using these theoretical techniques, they predicted conductance of different ferrocene complexes connected to Au electrodes via sulfur atoms attached to the cyclopenadienyl rings. 1,3-Ferrocene dithiolate (**1**) and 1,3′-ferrocene dithiolate (**2**) (Scheme 1) had similar calculated electron transmission coefficients with the 1,3 configuration having the highest conductivity at low bias. After these initial monomeric system calculations, they investigated ferrocene dimers and found that bisferrocene-2,4-dithiolate (**3**) had very similar predicted transmission coefficients to the 1,3′-ferrocene dithiolate (**2**).

**Scheme 2:**
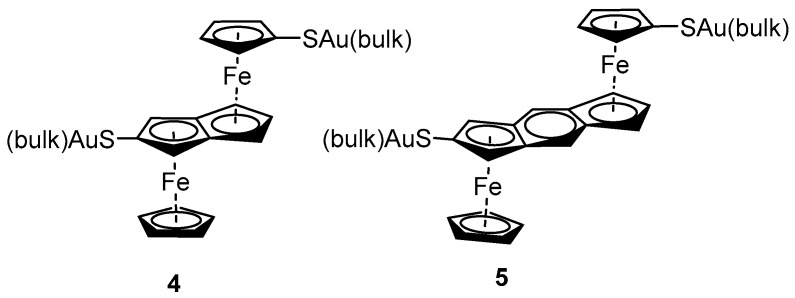
Bisferrocenylpentalenyl and bisferrocenylindacenyl dithiolates.

From here they moved to bis(ferrocenyl)pentalene-2,5-dithiolate (**4**) and *S*-(bisferrocenyl)indacene-2,6-dithiolate (**5**) (Scheme 2) which had much smaller HOMO–LUMO gaps (1.745 and 0.845 eV, respectively) compared with the 1,3–ferrocene dithiolate and bisferrocene-2,4-dithiolate (3.024 and 2.772 eV). The small gap for the *S*-(bisferrocenyl)indacene-2,6-dithiolate (**5**) results in small energy differences between transmission coefficient peaks above and below the Fermi level. The conclusion from this work was that both metal–metal separation distance and HOMO–LUMO gaps were both important factors in conductance properties of ferrocene-based oligomers. As of 2018, it does not appear that anyone has yet made any of these materials to test these predictions.

In 2011, Zhang and co-workers combined nonequilibrium Green’s function techniques with density functional theory to predict and compare charge transport properties of a series of double quantum dots formed by ferrocenes with saturated carbon and silicon bridges [Fc-(CH_2_)_n_]_2_ and [Fc-(SiH_2_)_n_]_2_ where n = 1, 2, 3 [10]. Only one of these species [Fc-SiH_2_]_2_ is predicted to be a rectifier. This predicted rectification is based on its transmission spectrum near the Fermi level, which correlates with higher current at negative bias than at positive bias, in addition to predicted HOMO–LUMO gaps, which are predicted to be narrower at negative bias (4.70 eV) than at positive bias (4.92 eV).

**Scheme 3:**
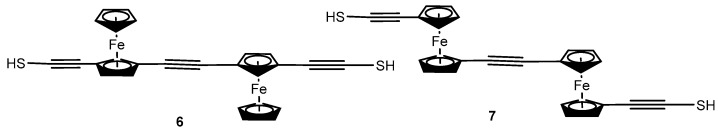
Thiolethynyl-Fc-ethynyl-Fc-thioethynes (Fc = ferrocenyl).

In 2017, Yuan and co-workers reported a model study predicting electron transport for 2 different thiolethynyl-Fc-ethynyl-Fc-thiolethynyl molecules (Fc = ferrocenyl) (**6**, **7**) (Scheme 3) if sandwiched between two electrodes [11]. While no rectification behavior was predicted, electron transport was predicted to be higher for the molecule with the central ethynyl group connecting cyclopentadienyl rings which did not also contain the thiolethynyl groups.

### 2.2. Experimental Work on Ferrocene-Containing Rectifiers

**Scheme 4:**
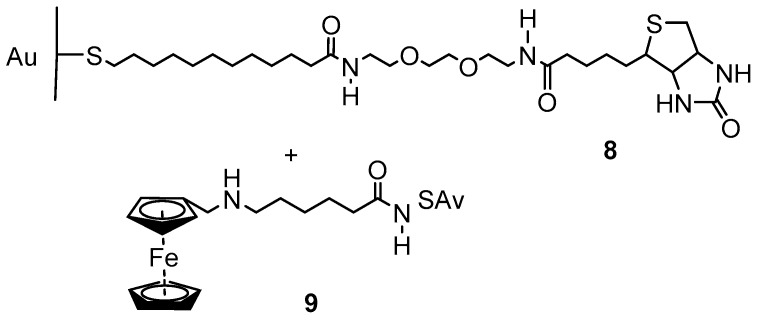
Bioconjugate rectifier components.

Azzaroni and co-workers reported construction of a ferrocene-containing bioconjugate that functions as a molecular rectifier in 2008 [12,13]. To prepare the final molecular rectifiers tested in this work, they first treated a gold electrode with a 9:1 ratio of 11-mercapto-1-undecanol and 12-mercaptododecanoic-(8-biotinoylamido-3,6-dioxaoctyl)amide. This produced a biotinylated electrode (**8**) which was then treated with ferrocene-labeled streptavidin (Fc-SAv) (**9**) (Scheme 4) (prepared by treating streptavidin (SAv) with *N*-(ferrocenylmethyl-6-amino)hexanoic acid, *N*-hydroxysuccinimide, and dimethylaminopropyl ethyl carbodiimide). This modified streptavidin (**7**) was determined to contain, on average, 14 ferrocenes per protein molecule. 

The electrochemical state of the ferrocenes incorporated into the protein layer could easily be controlled by electrode potential. Electrochemical cycling of the Au electrode in a solution of [Fe(CN)_6_]^4−^ (ferrocyanide) yielded an anodic peak and no cathodic peak since ferrocenium is spontaneously reduced to ferrocene by ferrocyanide whereas ferrocene is inert to [Fe(CN)_6_]^3−^ (ferricyanide). Using overnight incubation, the very specific and strong interactions between biotin and SAv resulted in a densely packed film on the electrode surface. This film mediated and rectified electron transfer between the redox donor (ferrocyanide) in solution and the Au electrode. This is the only example of a ferrocene-containing bioconjugate molecular rectifier that has appeared in the last 10 years. While not reporting after 2008 on bioconjugate molecular rectifiers, this group has subsequently reported use of hydrophobic lamellar domains as ferrocene hosts to create electrochemically active films [14].

**Scheme 5:**
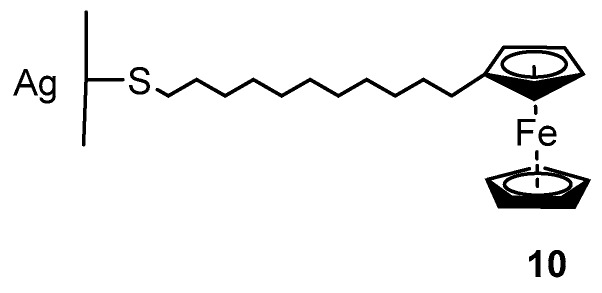
Ferrocenylundecanethiol on Ag.

There continue to be a number of reports of sulfur-anchored ferrocene-containing molecular rectifiers. The first from this last 10 year period are reports from the Whitesides group [15,16]. In this work they used 11-(ferrocenyl)-1-undecanethiol deposited onto ultraflat silver electrodes (**10**) (Scheme 5) embedded in cured optical adhesive and a eutectic alloy of gallium and indium (EGaIn) as the top electrode. They performed control experiments to show that alkanethiol junctions lacking the Fc moiety did not rectify and they also showed that top electrodes other than EGaIn also rectified currents with rectification ratios (R) of 10–100. They rationalized their large rectification ratios (R ≈ 130) by invoking use of the HOMO of ferrocene in charge transport at negative bias (hopping plus tunneling) but not at positive bias (charge transport by tunneling only).

**Scheme 6:**
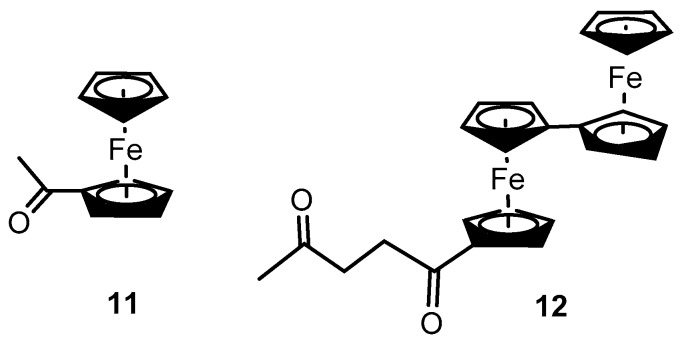
Acyl-containing ferrocenes.

Subsequent to this initial work by the Whitesides group, Nijhuis and co-workers and co-authors have continued to advance this research area. In 2010 they switched to an Au bottom electrode and first added heptathioether-functionalized β-cyclodextrin (βCD) to the Au to create a supramolecular platform on Au [17]. Dendrimers with termini functionalized with acetylferrocene (**11**) and 4-oxopentanoylbiferrocene (**12**) (Scheme 6) were adsorbed onto the platform and a dendrimer with an adamantane terminus was used as a control. The ferrocene junction incorporating (**11**) achieved an average R value of 7.7 whereas the one using bisferrocene (**12**) had an average R of 170. These authors again pointed to the importance of the ferrocene having a HOMO close to that of the Fermi levels of both electrodes (Au and EGaIn in this case).

In 2013, Thompson, Nijhuis, and co-workers reported that the number of CH_2_ groups between the sulfur and the ferrocene in the Ag–alkanethiol ferrocene–EGaIn rectifiers was a critical design element [18]. Odd numbers of CH_2_ units had more favorable van der Waals interactions—hence, lower packing energies—and this contributed to 10-times-better current rectification and about a 10% increase in working devices with also increased reproducibility. Maximum rectification (R ≈ 100) occurred with 11 CH_2_ units but 9 and 13 were also quite good and all were significantly better than 8, 10, and 12 CH_2_ units (R ≈ 10).

In 2016 these groups published a number of follow-on papers in this area, including a review of their work [19]. Some focused on transistors/charge transport across single-molecule junctions rather than rectifiers, so will not be covered by this review [20,21]. In this year, they reported a model of the limits of molecular tunneling rectification as temperature is changed. They also presented rectification data for alkanethiolates with the Fc unit close to Ag, close to EGaIn, and centered in between the 2 electrodes, and showed that their single-level transport model where R is plotted against the number of CH_2_ units between Ag and Fc fit the observed experimental data [22]. Separately, they also went back to the idea that 11 CH_2_ units between S and Fc were ideal and made a series of compounds with 10 CH_2′_s and then changed the one atom or group closest to the Fc; this, in turn, changed the tilt angle of the Fc unit relative to the EGaIn electrode and the rest of the linker chain [23]. The compounds with the smallest tilt angles were the original molecule with 11 CH_2′_s and the one where the last CH_2_ had been changed to NHC=O. Other groups that lead to larger tilt angles resulted in drastically lower R values. Most recently, Nijhuis and Veciana and co-workers have reported a dithiol rather than monothiol linker terminated in Fc, but also Fc attached to an additional electroactive head group—the polychlorotriphenylmethyl (PTM) group [24]. They found that when the PTM headgroup was in its electron-paired form it had high R (99), but when it was oxidized to the PTM radical, its R value dropped to 6; they attributed this to a change in the junction molecule molecular orbital that was being accessed predominantly for rectification. Most recently, Nijhuis and Barco have published a model for how to determine when molecules are noninteracting or interacting in tunnel junctions [25].

**Scheme 7:**
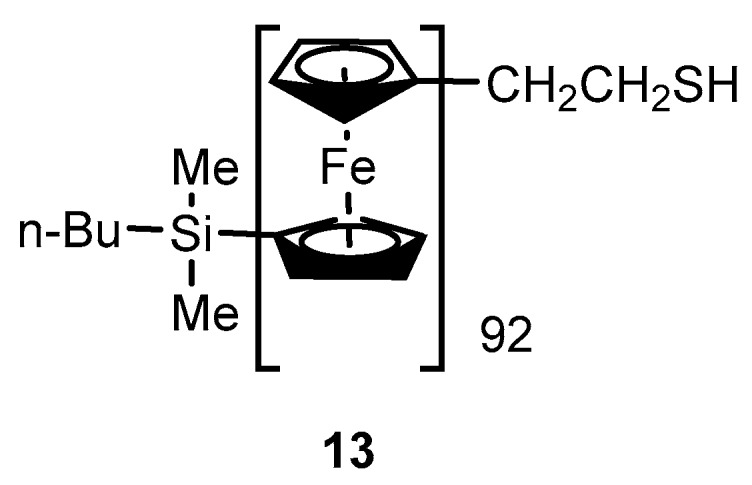
Sulfur end-functionalized poly(ferrocenyldimethylsilane).

In 2010, Mayer and co-workers reported using 6-ferrocenyl-1-hexanethiol (Fc-HT) in a Au bottom electrode/Fc–HT–Au tip junction with a reported RR of 20 [26]. In 2011, Vancso and Song reported that sulfur end-functionalized poly(ferrocenyldimethylsilane) (PFDMS) (**13**) (Scheme 7) could be grafted onto a gold electrode and that it showed current rectification in a solid electrode/electrolyte system using ferro/ferricyanide as electrolyte, but the magnitude of this rectification was not quantified [27].

**Scheme 8:**
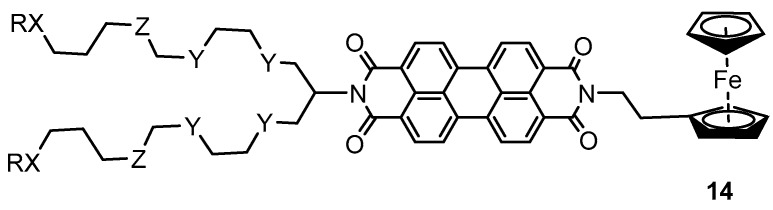
Swallowtail ferrocenes.

Mattern and co-workers reported a series of ferrocene-containing perylene bisimides having either polyethylene glycol (PEG) or sulfur swallowtail groups for monolayer ordering and electrode binding [28]. PEG swallowtails (Y = O, Z = OMe terminus) (**14**) (Scheme 8) were used for orienting Langmuir–Blodgett monolayers, and alkyl swallowtails with sulfur anchors (Y = Z = CH_2_, X = S) (**14**) were used with gold electrodes. 

**Scheme 9:**
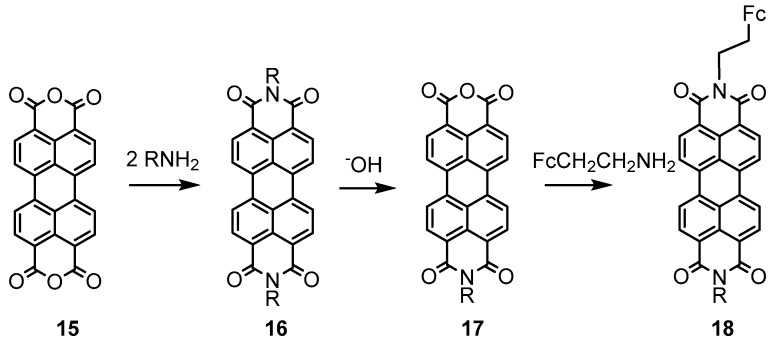
Preparation of swallowtail ferrocenes.

In general, these compounds were synthesized via a three-step sequence where 2 equivalents of swallowtail amine were added to perylenetetracarboxylic bisanhydride (**15**) (Scheme 9), followed by hydrolysis of one of the imide groups back to the anhydride (**17**). The mono anhydrides were then treated with ferrocenylethylamine to prepare ferrocene-containing imides (**18**). Yields for the final reactions to prepare compounds with PEG swallowtails were significantly lower (20–24%) than yields of reactions to prepare sulfur swallowtails (79–87%). Rectification behavior of these ferrocenes was not reported in this paper but the rectification behavior of the sulfur-swallowtail-containing molecule with XR = S–Acetyl was reported in 2016 [29]. This compound was chemically adsorbed onto a Au electrode and then contacted with an EGaIn electrode. Somewhat surprisingly, they noted that the direction of rectification was bias-dependent. At positive V (1.0V), the RR was 70–174; at higher voltages (2.5V), the rectification increased in the negative direction, reverse rectification ratio (RRR) = 3–35. They explained this reversal by noting that at positive EGaIn bias, the EGaIn electrode is the electron acceptor and the HOMO of the rectifier is most important. At negative EGaIn bias, that electrode is the electron donor, so the LUMO of the rectifier molecules becomes important.

**Scheme 10:**
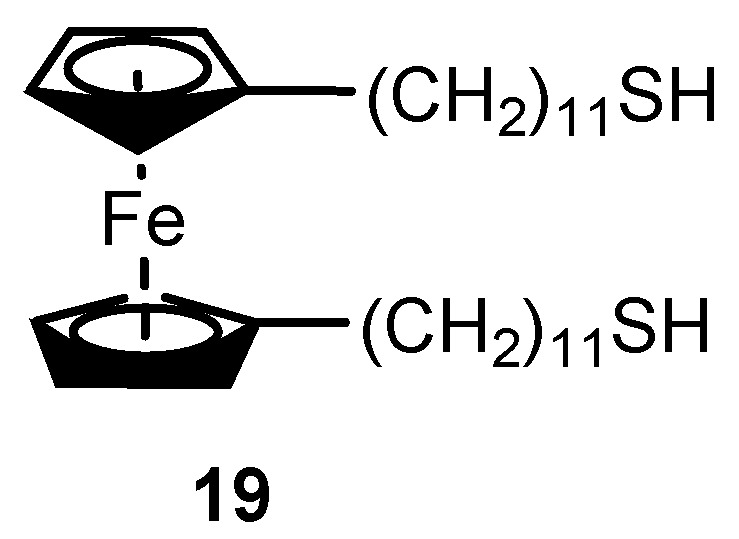
Ferrocenyl dithiol.

In 2013, Richter and co-workers reported a transistor containing a ferrocenyl dithiol (**19**) (Scheme 10) [30]. Surprisingly, they found that the gate voltage of the transistor containing this material could be tuned such that the transistor exhibited rectifying behavior.

**Scheme 11:**
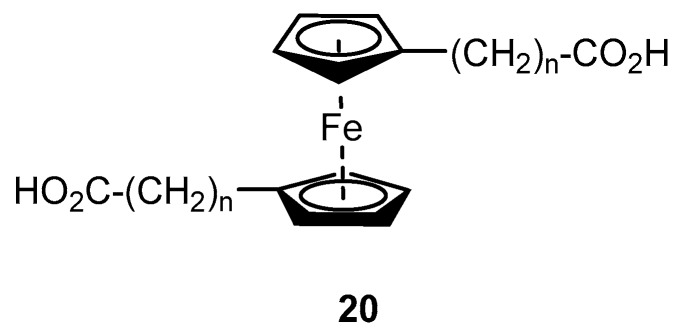
Ferrocene dicarboxylic acids.

In 2014, Zhou, Jin, and co-workers prepared a series of dicarboxylic acids containing ferrocenes (**20**) (Scheme 11) and they measured their conductances rather than rectification compared to non-ferrocene-containing dicarboxylic acids [31]. Perhaps as expected, measured conductance was much higher for the ferrocenes than for the alkyl dicarboxylic acids and the conductance for the shortest molecules (n = 0) was highest. Increased conductance was attributed to both a reduction in the tunneling barrier between electrodes and a smaller HOMO–LUMO gap when the ferrocene is present.

Also this year, Xiang, Lee, and co-workers reported electrodes prepared from ferrocenyl(CH_2_)_n_SH (where n = 6, 8 and 11) that exhibited temperature-dependent rectification behavior [32]. In addition to ferrocene deposition onto solid gold electrodes, they also reported preparation of rectifying electrodes onto a flexible polyimide substrate. In 2016, Lee and co-workers followed up on this report with a temperature-dependent transition voltage spectroscopy analysis of these electrodes and concluded that the origin of this temperature-dependent behavior was redox-induced conformational changes in the ferrocene alkanethiolates [33].

In 2017, Kwak and co-workers took the ferrocenylundecanethiol molecule largely studied previously by Nijhuis and co-workers and used it in a thin-layer electrochemical cell rather than an electrode as a programmable electrochemical rectifier [34]. Cells with a gap thickness between electrodes on the order of 4 μm showed R values up to 160.

**Scheme 12:**
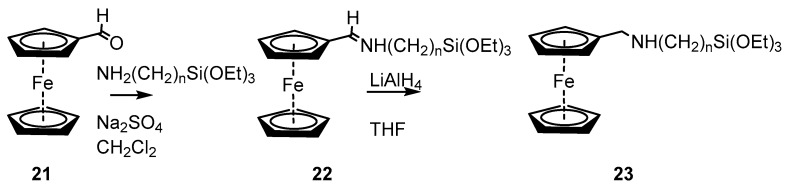
Synthesis of ferrocenylalkylsilanes.

Earlier this year from our lab, we reported the synthesis of four ferrocenylalkylsilanes (**22**, **23**, n = 3, 11) (Scheme 12) and their rectification behavior when sandwiched between silicon and EGaIn electrodes [35]. We wanted to study these molecules since they could be used with Si rather than Au or Ag bottom electrodes and their synthesis is simpler than that of the ferrocenylalkanethiols. We observed rectification ratios as high as 150 with these materials. In order to make devices which are compatible with the nearly ubiquitous methods used in silicon-based technologies, we chose to use a triethoxysilane anchoring group which covalently bonds to the native oxide present on a silicon wafer. The choice of this anchoring group facilitates the use of silicon wafers, which are nearly atomically flat as grown, with no additional fabrication steps [36]. In addition, the imine and amine ferrocene molecules (**22**, **23**) are obtained via a one- to two-step synthesis, in a fast and effective process.

Also this year, Zhang, Wang, and co-workers reported the preparation and testing of a number of rectifiers using the undecanethiolate linker to a Ag bottom electrode with the linker terminus being a variety of metallocenes including ferrocene [37]. Interestingly, spin filtering efficiency in the electrodes in addition to rectification could be obtained with the magnetic metallocene head groups they used (MCp_2_ where M = Cr, Mn, Co, Ni).

## 3. Conclusions

Use of ferrocenes as components in molecular junctions has been an area of intense work, both computational and experimental, over the last ten years. In particular, ferrocene-terminated alkane thiolates have been particularly well studied whereas ferrocenes with silane, carboxylate, and phosphonate linker groups have not been investigated in as much detail. Interestingly, ferrocenyl alkane chains that have sugar termini have also recently been reported but rectification behavior of electrodes that contain those molecules has not [38]. Ferrocene is particularly useful for work in this field since its HOMO is close in energy to the Fermi levels of typically used electrodes. Work on using metallocenes other than ferrocene in molecular rectifiers and diodes has started and can be expected to expand over the next ten years.
